# The Effect of Hepatitis B Infection on Levels of Fibrinogen, Protein C, and Protein S in Pregnant Women

**DOI:** 10.1155/jp/5239969

**Published:** 2026-03-08

**Authors:** Abiba Alhassan Khalifah, Stephen Twumasi, Allwell Adofo Ayirebi, Wina Ivy Ofori Boadu, Francis Agyei Amponsah, Joseph Frimpong, David Amoah Afrifah, Ernest Appiagyei, Albert Ntim Boadu, Daniel Nii Martey Antonio, Enoch Odame Anto

**Affiliations:** ^1^ Department of Medical Diagnostics, Faculty of Allied Health Sciences, College of Health Sciences, Kwame Nkrumah University of Science and Technology, Kumasi, Ghana, knust.edu.gh; ^2^ Garden City University, Kumasi, Ghana; ^3^ Kumasi South Hospital, Kumasi, Ghana

**Keywords:** albumin, aPTT, bilirubin, fibrinogen, hepatitis B, liver enzymes, pregnancy, protein C, protein S, PT

## Abstract

**Background:**

Viral hepatitis has been associated with profound alterations in the coagulation system as well as liver biomarkers. Meanwhile, during pregnancy, the coagulation system also undergoes significant changes with an increase in the majority of the clotting factors and a decrease in natural anticoagulants. This study is aimed at evaluating the coagulation profile and liver biomarkers among hepatitis B‐infected pregnant women in a Ghanaian population.

**Methods:**

This case–control study was conducted at Afrancho Polyclinic in the Ashanti Region, Ghana from January 2022 to July 2023. This study recruited 90 hepatitis B pregnant women as cases and 90 hepatitis B‐negative pregnant women as controls. A structured questionnaire was used to obtain sociodemographic, obstetric, and clinical data from each participant.

**Results:**

Levels of albumin, fibrinogen (4.09 [3.57–5.94] vs. 6.89 [5.43–9.08], *p* < 0.0001), protein C (2.46 [1.09–3.42] vs. 4.12 [2.96–6.07], *p* < 0.0001), and protein S (2.61 [2.20–3.36] vs. 2.98 [2.53–3.54], *p* = 0.036) were significantly reduced in the hepatitis B‐positive pregnant women than the negative controls. However, there were higher levels of AST, ALP, and bilirubins in hepatitis B‐positive pregnant women than the controls. Also, protein C and protein S had a significantly positive association with PT and aPTT, whereby a rise in protein C and protein S resulted in an increasing PT and aPTT, respectively (all *p* values < 0.05). Conversely, albumin had a negative correlation with both PT and aPTT (*p* value < 0.05). In a ROC analysis, aPTT had the highest area under the curve (AUC) value (AUC = 0.881) and the optimal clotting time at which aPTT indicated chronic hepatitis B was ≥ 35.7 s with sensitivity of 79.4% and specificity of 91.6%.

**Conclusion:**

Pregnant women with hepatitis B infection present with significant changes in their coagulation parameters, natural anticoagulants, and liver biomarkers. Furthermore, fibrinogen, protein C, and aPTT showed accurate diagnostic potential in detecting chronic viral hepatitis B infection and may be valuable surrogate indicators for managing chronic hepatitis‐related complications.

## 1. Introduction

Viral hepatitis is a critical global health issue, with infected patients facing a substantial risk of morbidity and mortality. The two most common hepatotropic viruses that cause chronic hepatitis are hepatitis B and C viruses, infecting more than 360 million people globally, with over 1 million deaths [1]. In 2020, the African region represented 26% of worldwide hepatitis B and C cases and caused 125,000 related fatalities, as per the WHO [2]. Ghana faces hyperendemic hepatitis B viral (HBV) infection, with an estimated prevalence ranging from 8.36% (2020) to 12.30% (2016). A recent systematic review reported 7.4% as HBV prevalence in pregnant women [3]. Also, a recent study in Nigeria recorded 8% prevalence of HBV in pregnant women [4]. Transmission of HBV from infected mothers to their newborns, during pregnancy or childbirth, plays a significant role in sustaining the pool of chronically infected individuals worldwide [5].

The liver synthesizes vital proteins for both coagulation (procoagulants) and anticoagulation purposes [6]. In the advent of chronic HBV infection, there is activation of hepatic stellate cells, resulting in the replacement of healthy hepatic parenchyma with excess extracellular matrix, termed as liver fibrosis, which can advance to a more severe form known as cirrhosis [7].

Because the liver is so central in the coagulation process, patients with liver fibrosis and cirrhosis display some profound changes in the components of the coagulation cascade [8]. Levels of procoagulant proteins such as fibrinogen and others are decreased, with a concomitant decrease in anticoagulant proteins such as protein C and protein S in liver fibrosis and cirrhosis subjects. This causes a state of delicate hemostatic balance, which increases the risk of bleeding or thrombosis to such patients [8].

Studies have also reported prolongation of prothrombin time (PT), international normalized ratio (INR), and activated partial thromboplastin time (aPTT) following viral hepatitis with dysfibrinogenemia and thrombocytopenia [9]. This is because of the loss of the integrity of both the extrinsic pathway of coagulation and the factors of the common pathway. And the degree of prolongation of this clotting time has been suggested to correlate with the degree of deficiency or inhibition of extrinsic or common pathway clotting factors; hence, the severity of the liver disease [7].

Normally during pregnancy, changes are often seen in the hepatic biochemical profile. Usually, there is elevation in the level of serum alkaline phosphatase (ALP), and this elevation may be up to 2–4 times the normal baseline level. This is because the placenta produces additional ALP during pregnancy, whereas the serum albumin usually drops, and this is attributed to the total plasma volume. However, the serum levels of aspartate amino transferase (AST), alanine amino transferase (ALT), and bilirubin usually remain normal, and any elevation seen may be attributed to a liver condition [10]. AST and ALT are often released into the bloodstream once there is a hepatocellular damage, so AST and ALT serum level elevation correlate more with hepatic injury [11].

Several studies have reported an increased risk for adverse pregnancy outcomes in pregnancies associated with maternal HBV infection [12]. HBV infection has also been linked to elevated levels of liver function test (LFT) markers, as well as significant alterations in the proteins of the coagulation cascade [8]. Pregnancy also predisposes a woman into a hypercoagulable state, and therefore, a pregnant woman coupled with HBV infection can be at a much greater risk for liver markers and coagulation abnormalities [13, 14]. In Ghana, although a positive HBV carrier status is common among pregnant women, little is known about the impact of the infection on their liver markers and hemostatic profile. Therefore, this study sought to determine hepatitis B infection′s impact on fibrinogen, protein C, protein S levels, and liver markers in pregnant women.

## 2. Materials and Methods

### 2.1. Study Design, Study Duration, and Study Site

This hospital‐based case–control study was carried out from January 2022 to July 2023.

This research was conducted at Afrancho Polyclinic in the Afigya Kwabre South District, Ashanti Region, Ghana. The district lies in the center of the Ashanti Region and covers an area of about 159 km^2^. Its proximity to the urban center of Kumasi has driven rapid population growth, transforming the district into a vibrant residential area characterized by expanding and dynamic settlements. According to the 2021 population census by the Ghana Statistical Service, the district has a total population of about 234,000. Afrancho Polyclinic offers a wide range of medical services that are categorized under units and departments such as; antenatal clinic, emergency unit, laboratory department, eye clinic, theater unit, labor unit, mental health department, disease control unit, out‐patient department, medical imaging, and others.

### 2.2. Ethical Consideration and Informed Consent

Ethical approval was obtained from the Committee on Human Research, Publication, and Ethics, School of Medical Sciences, Kwame Nkrumah University of Science and Technology (CHRPE/AP/834/22), as well as the management of Afrancho Polyclinic. The ethical approval was fixed for 1 year from 19th December, 2022, to 18th December, 2023. Also, both oral and written consents from all participants were obtained before their recruitment. All participants had the age to consent.

### 2.3. Sample Size Calculation

The research utilized the Kelsey′s formula to obtain the required sample

Ncases−Kelsey=r+1÷r P1−PZα+Zβ÷22÷p12−p2, and P=p12+r∗p÷r+1

Where the ratio of hepatitis B subjects to healthy controls is represented by “*r*”, which is 1:1 in the present study. The critical value of the normal dispersion at *α*/2 was represented as Z_*α*/2_. In this present study, with confidence interval of 95%, *α* is 0.05 and the critical value is 1.96. The critical value is of the normal distribution at *β* was represented by Z*β*. The study used a power of 80%, where *β* is 0.2, and the critical value is 0.84.

p1 represented the percentage of pregnant women living with hepatitis B in Ghana, which is 10.2%, whereas p2 represented the percentage of hepatitis B‐infected individuals in the control group, which is 6.8% according to Adjei et al. [15]. Subtracting p2 from p1 produced the minimal difference in proportions that is important medically.

The study therefore required a minimum of 54 pregnant women with hepatitis B, with 54 healthy controls. This study, however, enrolled a total of 180 subjects. Ninety hepatitis B‐positive subjects were selected as cases and 90 Hepatitis B pregnant women as controls.

### 2.4. Study Population and Selection of Participants

#### 2.4.1. Inclusion Criteria

**Figure 1:**
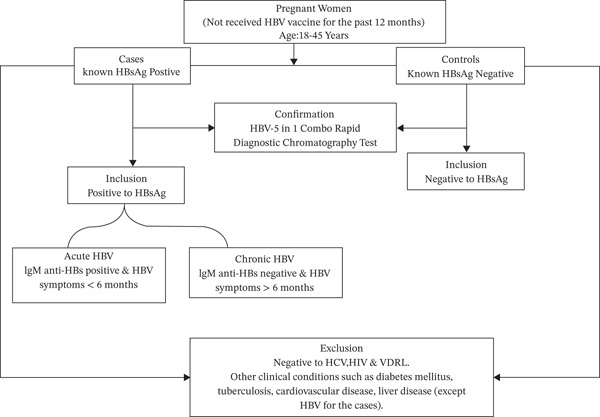
Flow chart depicting the inclusion and exclusion criteria of the study.

The participants were pregnant women visiting the antenatal clinic at different stages of gestation and were between the ages of 18 and 45 years. Sociodemographic data were obtained with a standardized questionnaire. Their clinical history and obstetric information were also obtained from their antenatal folder. Pregnant women who have been diagnosed with hepatitis B were selected as cases. Confirmation of the HBV was done with the rapid chromatographic method (Smartcare, China). The cases were further categorized into acute and chronic hepatitis B group based on the duration of infection and the presence or absence of IgM Hepatitis B core antibody (IgM anti‐HBc). The control group was also selected after participants were found to be negative to HBV and any other viral infection (Figure 1).

#### 2.4.2. Exclusion Criteria

For the cases, pregnant women showing any underlying chronic illness such as diabetes mellitus, tuberculosis, cardiovascular (including preeclampsia), or any other clinical condition other than hepatitis B infection were excluded from the study. Also, individuals with liver fibrosis or cirrhosis were excluded based on their fibrosis scores (Fibrosis‐4 [FIB‐4] and aspartate aminotransferase‐to‐platelet ratio index [APRI]).

Pregnant women who were reactive to HCV, HIV, and VDRL or any other clinical condition were also excluded. Also, HBV individuals were excluded under the control group. In addition, participants were excluded if they have received hepatitis B vaccination within the preceding 12 months. This was to avoid confounding the seroprevalence results. Recent vaccination may elevate hepatitis B antibody levels; therefore, this criterion ensures that detected antibodies reflect prior exposure rather than recent immunization, providing a more accurate estimate of natural seroprevalence [4] (Figure 1).

### 2.5. Sample Collection

Each subject′s venous blood (8 mL) was aseptically collected via the standard vacutainer system. About 4 mL was dispensed into a citrated anticoagulant bottle and gently mixed properly. Two mL was dispensed into a gel tube for LFT, and the remaining 2 mL was dispensed into an EDTA tube for full blood count. The citrated blood samples were immediately spun (2000 rpm for 20 min). Thereafter, portions of the platelet‐poor plasma (PPP) were aliquoted and stored at −80°C for later analysis of fibrinogen, protein C, and protein S. PT and aPTT were run the same day with the remaining PPP. The gel tube blood sample was centrifuged once clotted, and the serum was used for the LFT.

### 2.6. Laboratory Investigations

Full blood count was done with the Sysmex KX‐21N hematology analyzer (Sysmex Corporation, Japan). The hepatitis B profile status of the cases (HBsAg‐positive) was ascertained using an HBV‐5 in 1 Combo Rapid diagnostic Test from Smartcare, China. This HBV 5‐parameter Rapid Test Kit is a lateral flow chromatographic immunoassay for the qualitative detection of HBsAg, HBsAb, HBeAg, HBeAb, and HBcAb in human serum/plasma. The PT and aPTT were assessed by the manual method with strict adherence to standard operating procedures and quality control protocols, with reagents from Fortress Diagnostics Limited, United Kingdom. IgM anti‐HBc, fibrinogen, protein C, and protein S concentrations were measured by a solid‐phase Sandwich ELISA technique, using kits from Melson Shanghai Chemical Limited, China. All assays were performed according to the manufacturer′s instructions, with appropriate quality control measures to ensure accuracy and reliability. All kits were within expiry and validated by internal controls. The LFTs were run using Sinnowa semiautomated chemistry analyzer, with reagents from Fortress Diagnostics, United Kingdom. Concentrations of liver parameters such as liver enzymes (AST, ALT, ALP, and gamma glutamyl transferase [GGT]), bilirubin (total, direct, and indirect), total protein, albumin, and globulin were all estimated, with strict adherence to the manufacturer′s protocol.

### 2.7. Definition of Acute and Chronic Hepatitis B Infection

Acute hepatitis B infection was defined as hepatitis B virus infection, evidenced by the presence of HBsAg for less than 6 months, accompanied by detectable IgM anti‐HBc. Chronic hepatitis B infection was defined as a persistent hepatitis B virus infection, indicated by the continued presence of HBsAg for more than 6 months and the absence of IgM anti‐HBc. [14, 16].

### 2.8. FIB‐4 and APRI Scores

To exclude patients with liver fibrosis and cirrhosis, APRI and FIB‐4 scores were calculated. These fibrosis scores were derived using standard formulas: FIB‐4 = (age × AST) / (platelet 206 count × ALT^1/2^) and APRI = ([AST / ULN of AST] / platelet count) × 100, with the ULN for AST defined as 40 IU/L. Predefined thresholds with a specificity of 90% were applied as reference cutoffs (APRI ≥ 1.74 and FIB‐4 ≥ 1.90 for significant fibrosis; APRI ≥ 2.00 and FIB‐4 ≥ 2.31 for cirrhosis) [17]. Participants with fibrosis scores exceeding these thresholds were excluded from the study.

### 2.9. Statistical Analysis

Data were entered and cleaned using Microsoft Excel 2016 (Microsoft Corporation, Redmond, Washington, United States) and analyzed using IBM SPSS Statistics Version 26 (IBM Corporation, Armonk, New York, United States). Categorical data were represented as counts and proportions and subjected to comparison using either Chi‐squared or Fisher′s exact test. Logistic regression was used to assess how the sociodemographic characteristics predict HBV infection. Normality check was performed using Kolmogorov–Smirnov test. We represented nonnormally distributed data as median (interquartile range) and subjected them to comparison using Kruskal–Wallis test. Spearman′s correlation was utilized to determine the association of albumin, fibrinogen, fibrinogen/albumin ratio (FAR), protein C, and protein S levels with PT or APTT among HBV‐infected pregnant women. Receiver operating characteristics (ROC) curve of aPTT, PT, Fibrinogen, protein C, FAR, albumin, and protein S for detecting chronic viral hepatitis B infection were drawn and respective areas under the curve (AUCs) values were estimated. *p* < 0.05 signified statistical significance.

## 3. Results

### 3.1. Sociodemographic Characteristics Among the Study Participants

Age categories were evenly distributed (*p* = 0.456), with 47.2% among the control cohort and 52.8% among the HBV cohort aged 18–29. Marital status (*p* = 0.588), employment status (*p* = 0.465), residence (*p* = 0.709), religion (*p* = 0.594), ethnicity (*p* = 0.478), gravidity (*p* = 0.709), parity (*p* = 0.891), sickling status (*p* = 0.225), G6PD (*p* = 0.943), and blood group (*p* = 0.52) did not differ between the two cohorts.

However, a few notable differences were observed. The HBV group had a slightly higher percentage of individuals who were cohabiting (48.5%) compared with the control group (41.9%). Additionally, higher percentages of the HBV group were self‐employed (54.9% vs. 45.1%) and had tertiary education (64.3% vs. 35.7%). These differences, however, did not attain statistical significance (all *p* > 0.05).

Alcohol consumption and contraceptive use were significantly related to HBV infection. The HBV group had a higher proportion of individuals who reported alcohol consumption (75.0% vs. 25.0%) and contraceptive use (60.0% vs. 40.0%) compared with controls. The crude odds ratio (cOR) for alcohol consumption was 3.3 (95% CI: 1.0–10.7), indicating a positive association. Similarly, the cOR for contraceptive use was 2.3 (95% CI: 1.2–4.1), suggesting an increased likelihood of HBV infection among contraceptive users (Table 1).

**Table 1:** Association between sociodemographic characteristics and hepatitis B infection.

Variable	Controls (*N* = 90)	HBV (*N* = 90)	cOR (95% CI)	*p*
Age categories				0.456
18–29	42 (47.2)	47 (52.8)	Ref [18]	
30–42	48 (52.7)	43 (47.3)	1.2 (0.7–2.2)	
Marital status				0.588
Single	6 (50.0)	6 (50.0)	Ref [18]	
Married	70 (51.5)	66 (48.5)	0.9 (0.3–3.0)	
Cohabiting	13 (41.9)	18 (48.5)	1.4 (0.4–5.30	
Employment status				0.465
Unemployed	46 (53.5)	40 (46.5)	Ref [18]	
Self‐employed	37 (45.1)	45 (54.9)	1.4 (0.8–2.6)	
Formally employed	7 (58.3)	5 (41.7)	0.8 (0.2–2.8)	
Educational level				0.17
None	12 (41.4)	17 (58.6)	0.4 (0.1–1.2)	
Primary	15 (65.2)	8 (34.8)	0.9 (0.4–2.2)	
Junior	25 (43.9)	32 (56.1)	0.5 (0.2–1.3)	
Secondary	33 (57.9)	24 (42.1)	1.3 (0.3–4.8)	
Tertiary	5 (35.7)	9 (64.3)	Ref [18]	
Residence				0.709
Peri‐urban	19 (52.8)	17 (47.2)	0.9 (0.4–1.8)	
Urban	71 (49.3)	73 (50.7)	Ref [18]	
Religion				0.594
Christianity	71 (51.1)	68 (48.9)	0.8 (0.4–1.7)	
Islam	19 (46.3)	22 (53.7)	Ref [18]	
Ethnicity				0.478
Akans	51 (50.5)	50 (49.5)	0.6 (0.3–1.1)	
Ewe/Fante/Bono	4 (33.3)	8 (66.7)	0.5 (0.1–1.8)	
Northerners	35 (52.2)	32 (47.8)	Ref [18]	
Gravidity				0.709
Primigravida	19 (52.8)	17 (47.2)	0.9 (0.4–1.8)	
Multigravida	71 (49.3)	73 (50.7)	Ref [18]	
Parity				0.891
Nulliparous	22 (48.9)	23 (51.1)	1.0 (0.5–2.1)	
Primiparous	30 (52.6)	27 (47.4)	0.9 (0.4–1.7)	
Multiparous	38 (48.7)	40 (51.3)	Ref [18]	
Smoking status				0.316
No	90 (50.3)	89 (49.7)	Ref [18]	
Yes	0 (0.0)	1 (100.0)	> 100 (0‐inf)	
Alcohol consumption				**0.036**
No	86 (52.4)	78 (47.6)	Ref [18]	
Yes	4 (25.0)	12 (75.0)	3.3 (1.0–10.7)	
Contraceptive use				**0.007**
No	54 (60.0)	36 (40.0)	Ref [18]	
Yes	36 (40.0)	54 (60.0)	2.3 (1.2–4.1)	

*Note:* Data are shown as frequency and percentages; analyzed with Chi‐squared/Fisher′s test and logistic regression. *p* < 0.05 signified statistical significance. Bold values show statistical significance.

Abbreviations: cOR, crude odds ratio; HBV, hepatitis B viral infection; N, number.

### 3.2. LFT Markers Among the Study Groups

Serum levels of AST (36.4 [32.8–47.5] vs. 30.7 [26.4–39.2], *p* = 0.014), ALP (155.4 [138.3–304.5] vs. 115.4 [105.2–141.9], *p* < 0.0001), total bilirubin (5.6 [2.7–6.3] vs. 1.3 [1.0–1.9], *p* < 0.0001), direct bilirubin (1.7 [1.0–2.5] vs. 0.5 [0.3–0.8], *p* < 0.0001), and indirect bilirubin (3.3 [1.6–4.5] vs. 0.9 [0.6–1.1], *p* < 0.0001) were markedly elevated among acute HBV‐infected pregnant women relative to the control women. Similarly, serum levels of AST (37.3 [32.0–45.6] vs. 30.7 [26.4–39.2], *p* < 0.0001), ALP (132.4 [116.6–197.4] vs. 115.4 [105.2–141.9], *p* = 0.004), GGT (30.9 [24.5–37.6] vs. 27.2 [19.4–32.1], *p* = 0.007), total bilirubin (2.4 [1.8–4.3] vs. 1.3 [1.0–1.9], *p* < 0.0001), direct bilirubin (0.9 [0.6–1.8] vs. 0.5 [0.3–0.8], *p* < 0.0001), and indirect bilirubin (1.5 [1.1–2.5] vs. 0.9 [0.6–1.1], *p* < 0.0001) were markedly raised in chronic HBV‐infected women compared with those uninfected. However, these liver function indicators did not differ between the acute and chronic subjects (all *p* > 0.05). Fibrosis scores (FIB‐4 and APRI) were comparable among all groups (all *p* > 0.05). (Table 2).

**Table 2:** Liver function test markers among the study groups.

Variables	Controls (*N* = 90)	Acute HBV (*N* = 14)	Chronic HBV (*N* = 76)	Acute HBV vs. controls	Chronic HBV vs. controls
AST (U/L)	30.7 (26.4–39.2)	36.4 (32.8–47.5)	37.3 (32.0–45.6)	**0.014**	**< 0.0001**
ALT (U/L)	28.6 (22.5–36.4)	32.3 (24.6–37.0)	23.4 (22.5–38.9)	NS	NS
ALP (U/L)	115.4 (105.2–141.9)	155.4 (138.3–304.5)	132.4 (116.6–197.4)	**< 0.0001**	**0.004**
GGT (U/L)	27.2 (19.4–32.1)	32.7 (18.3–37.6)	30.9 (24.5–37.6)	0.238	**0.007**
Total bilirubin (mg/dL)	1.3 (1.0–1.9)	5.6 (2.7–6.3)	2.4 (1.8–4.3)	**< 0.0001**	**< 0.0001**
Direct bilirubin (mg/dL)	0.5 (0.3–0.8)	1.7 (1.0–2.5)	0.9 (0.6–1.8)	**< 0.0001**	**< 0.0001**
Indirect bilirubin (mg/dL)	0.9 (0.6–1.1)	3.3 (1.6–4.5)	1.5 (1.1–2.5)	**< 0.0001**	**< 0.0001**
Total protein (g/dL)	6.1 (5.7–6.5)	5.6 (5.1–6.3)	5.9 (5.5–6.5)	NS	NS
Globulin (g/dL)	2.6 (2.3–2.8)	2.8 (2.1–3.1)	2.7 (2.2–3.1)	NS	NS
FIB‐4	0.3 (0.1–1.0)	0.3 (0.1–0.6)	0.3 (0.1–1.4)	NS	NS
APRI	0.3 (0.2–0.9)	0.4 (0.3–0.9)	0.4 (0.2–0.8)	NS	NS

*Note:* Data shown as median (interquartile range); analyzed with Kruskal–Wallis test *p* < 0.05 signified statistical significance. Bold values show statistical significance.

Abbreviations: ALP, alkaline phosphatase; ALT, alanine transaminase; APRI, aspartate aminotransferase‐to‐platelet ratio index; AST, aspartate transaminase; FIB‐4, fibrosis 4; GGT, gamma glutamyl transferase, HBV; hepatitis B viral infection; N; number; NS, not significant.

### 3.3. Comparison of PT, aPTT, INR, and Albumin Among the Study Groups

PT (17.5 [16.4–19.5] vs. 16.1 [15.0–18.4] vs. 14.1 [13.1–15.7], *p* < 0.0001) and INR (1.55 [1.42–1.78] vs. 1.38 [1.27–1.65] vs. 1.17 [1.06–1.34], *p* < 0.0001) were significantly raised among acute HBV, followed by those with chronic HBV, and then the controls (HBV‐negative pregnant women). aPTT was significantly elevated in both acute and chronic HBV compared with the HBV‐negative pregnant women (*p* < 0.0001). However, aPTT was similar between acute and chronic HBV (*p* > 0.05). Moreover, albumin was significantly lower in both acute (31.0 [28.0–36.0] vs. 36.0 [31.0–39.0], *p* = 0.005) and chronic HBV (33.0 [29.0–37.0] vs. 36.0 [31.0–39.0], *p* = 0.036) relative to the HBV‐negative pregnant women but did not differ between the acute and chronic HBV group (*p* > 0.05) (Figures 2a, 2b, 2c, and 2d).

Figure 2(a–d) Comparison of PT, aPTT, INR, and albumin among the study groups. PT, prothrombin time; aPTT, activated partial thromboplastin time; INR; international normalized ratio. (e–h): Comparison of levels of fibrinogen, fibrinogen/albumin ratio (FAR), protein C, and protein S among the subjects.

**Figure:**
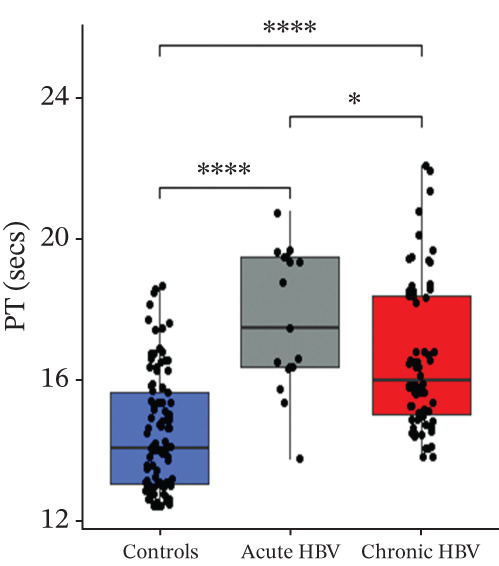
(a)

**Figure:**
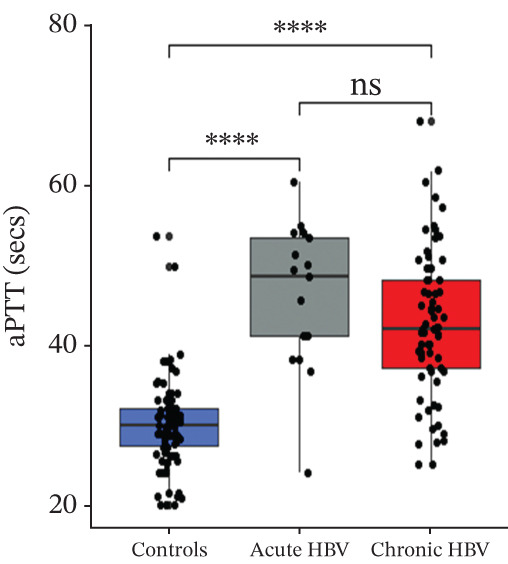
(b)

**Figure:**
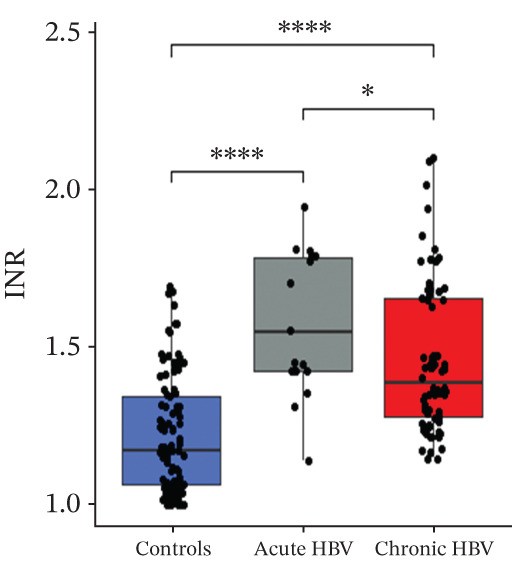
(c)

**Figure:**
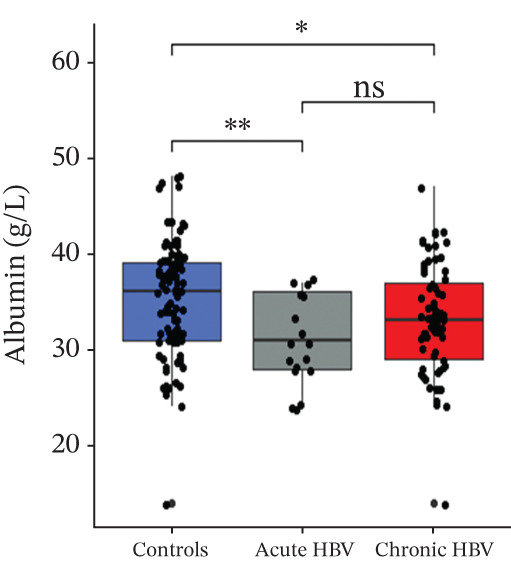
(d)

**Figure:**
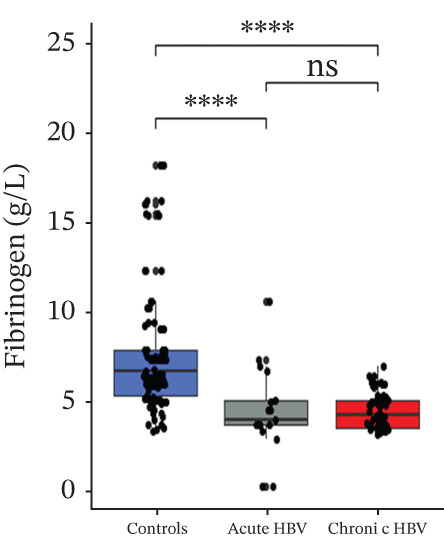
(e)

**Figure:**
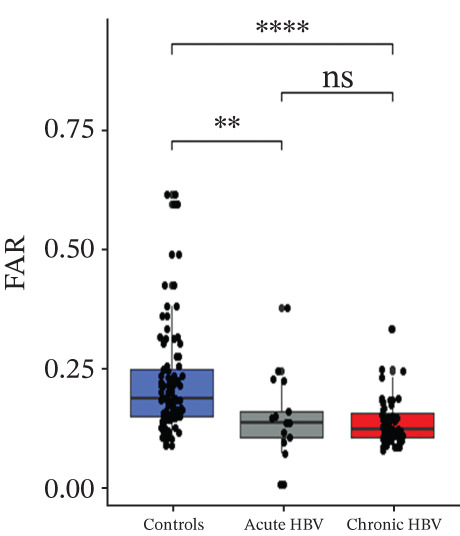
(f)

**Figure:**
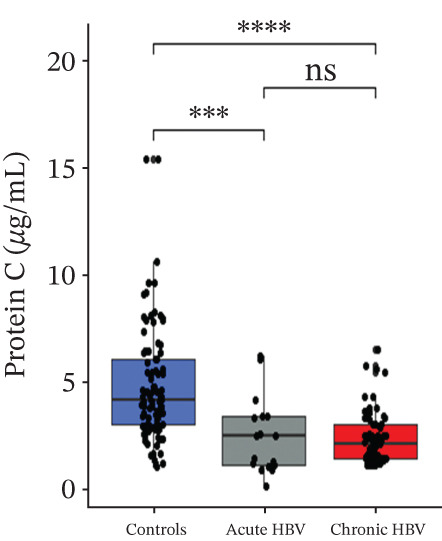
(g)

**Figure:**
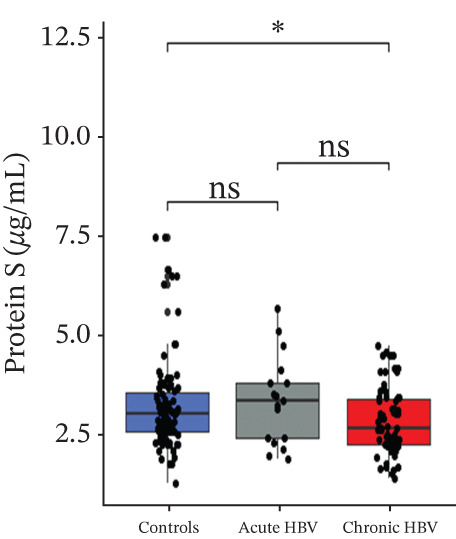
(h)

### 3.4. Comparison of Levels of Fibrinogen, FAR, Protein C, and Protein S Among the Subjects

Levels of fibrinogen (4.09 [3.57–5.94] vs. 6.89 [5.43–9.08], *p* < 0.0001), FAR (0.14 [0.11–0.20] vs. 0.19 [0.17–0.26], *p* = 0.002), and protein C (2.46 [1.09–3.42] vs. 4.12 [2.96–6.07], *p* < 0.0001) were significantly reduced among the acute relative to the HBV‐negative pregnant women. Again, fibrinogen (4.33 [3.60–5.20] vs. 6.89 [5.43–9.08], *p* < 0.001), FAR (0.13 [0.11–0.17] vs. 0.19 [0.17–0.26], *p* < 0.0001), and protein C (2.10 [1.45–3.00] vs 4.12 [2.96–6.07], *p* < 0.0001) were significantly reduced among the chronic HBV relative to the HBV‐negative pregnant women. However, their levels were similar between the acute and chronic HBV groups (all *p* > 0.05). Also, protein S (2.61 [2.20–3.36] vs. 2.98 [2.53–3.54], *p* = 0.036] was significantly reduced among the chronic HBV group relative to the control group but was similar between either the chronic and acute HBV or the acute and controls (Figures 2e, 2f, 2g, and 2h).

### 3.5. Association Between the Levels of Albumin, Fibrinogen, FAR, Protein C, and Protein S, in Comparison With PT and aPTT Among HBV‐Infected Pregnant Women

In a multivariate linear regression model, increasing FAR (*R* = 0.288, *p* = 0.006) and protein C (*R* = 0.273, *p* = 0.009) were associated with a significant increase in PT. However, in comparison with aPTT, these increments were not statistically significant. Meanwhile, protein S (*R* = 0.147, *p* = 0.046) was the only marker that had a positive and significant association with aPTT. In contrast, decreasing albumin (*R* = −0.279, *p* = 0.008), (*R* = −0.571, *p* < 0.001) was associated with significant increases in PT and aPTT, respectively. (Figures 3a, 3b, 3c, 3d, 3e, 3f, 3g, 3h, 3i, and 3j).

Figure 3(a–e): Association between the levels of albumin, fibrinogen, fibrinogen/albumin ratio (FAR), protein C, protein S, and prothrombin time (PT) among HBV infected pregnant women (f–j): Association between the levels of albumin, fibrinogen, FAR, protein C, protein S, and activated partial thromboplastin time (aPTT) among HBV infected pregnant women.

**Figure:**
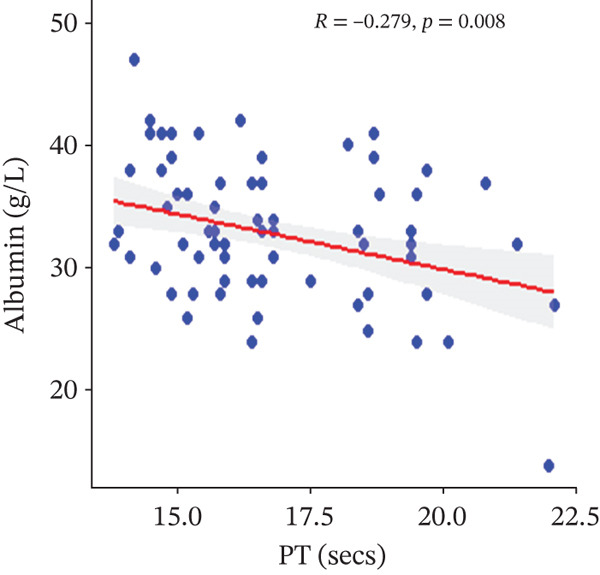
(a)

**Figure:**
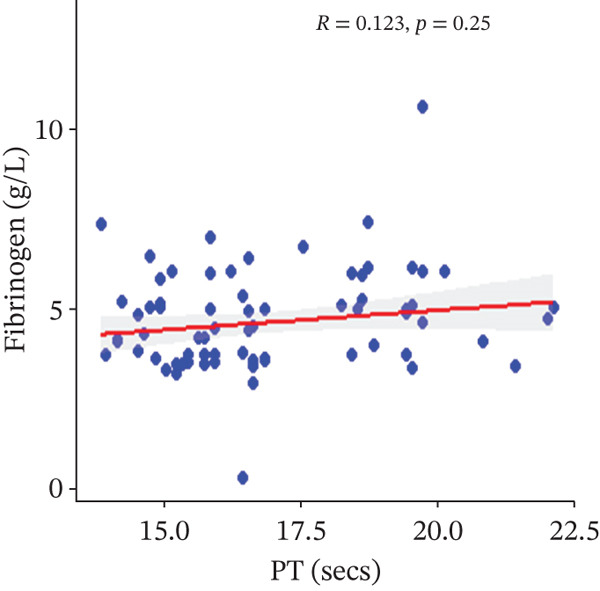
(b)

**Figure:**
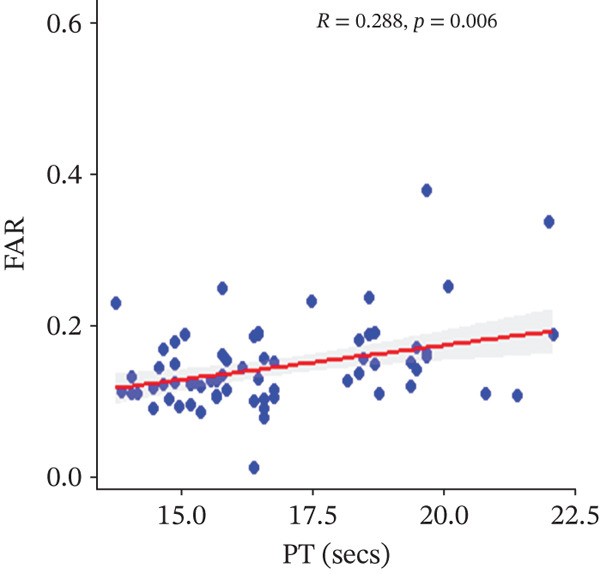
(c)

**Figure:**
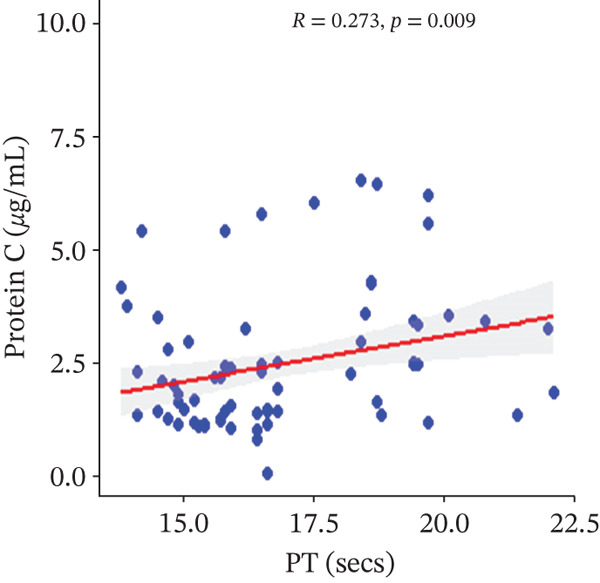
(d)

**Figure:**
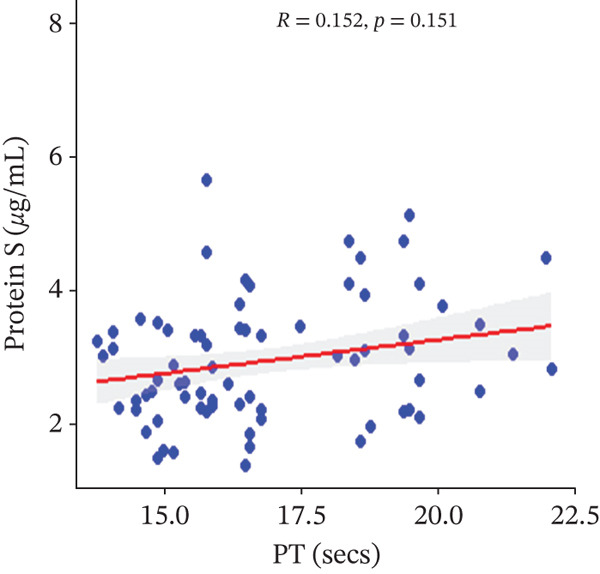
(e)

**Figure:**
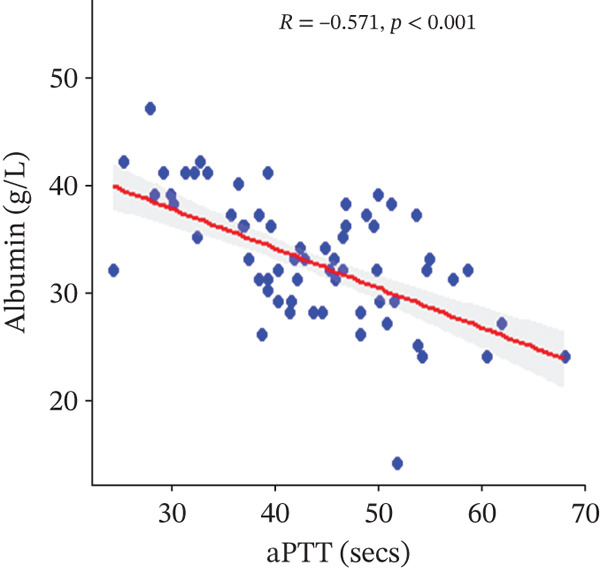
(f)

**Figure:**
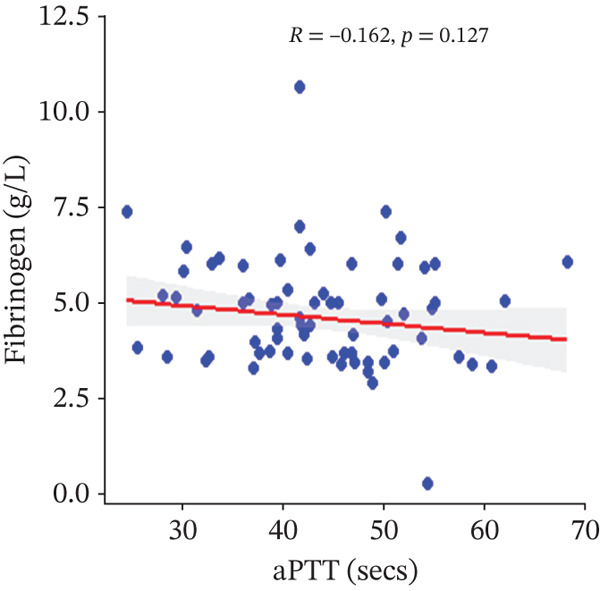
(g)

**Figure:**
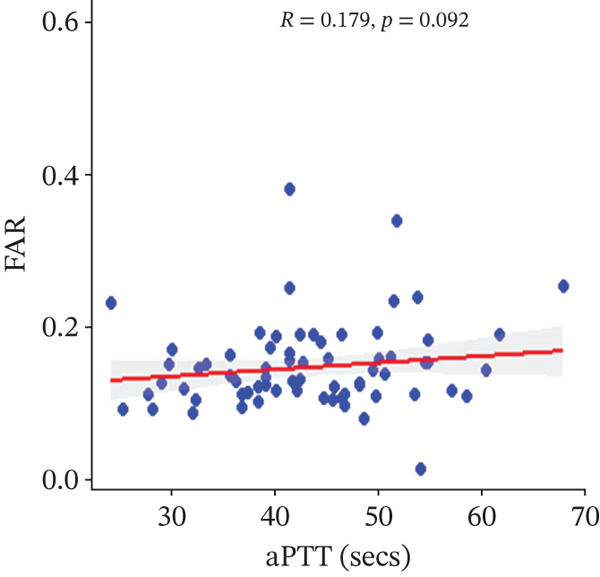
(h)

**Figure:**
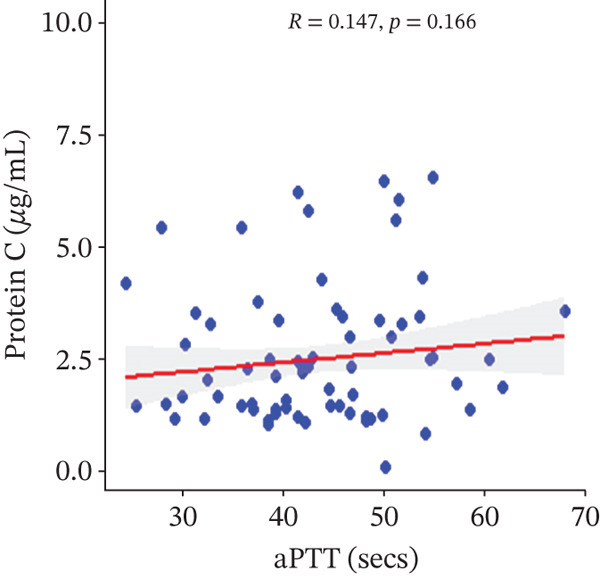
(i)

**Figure:**
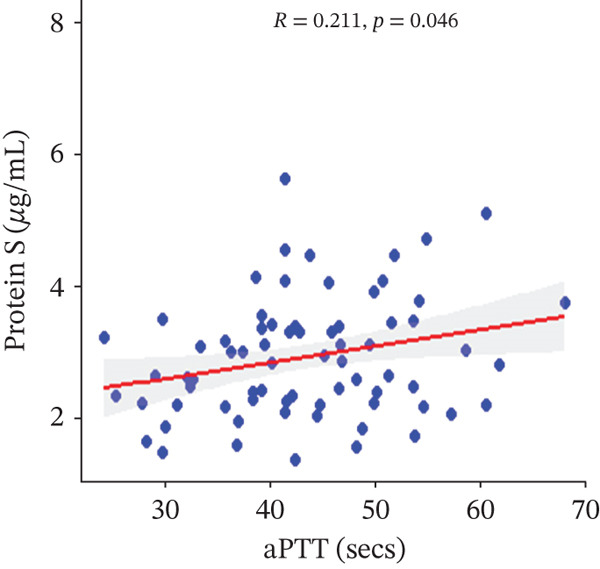
(j)

### 3.6. ROC Curve of aPTT, PT, Fibrinogen, Protein C, FAR, Albumin, and Protein S as Potential Indicators of Chronic Viral Hepatitis B Infection

In a ROC analysis, aPTT (AUC = 0.881), fibrinogen (AUC = 0.867), and protein C (AUC = 0.816) indicate chronic HBV with a very high AUC values. FAR (AUC = 0.793) and PT (AUC = 0.786) could also indicate chronic HBV with high AUC. However, albumin (AUC = 0.615) and protein S (AUC = 0.593) could poorly indicate chronic HBV (Figures 4a,b).

Figure 4(a–b) Receiver operating characteristics (ROC) curve of aPTT, PT, fibrinogen, protein C, FAR, albumin, and protein S as potential indicators of chronic viral hepatitis B infection.

**Figure:**
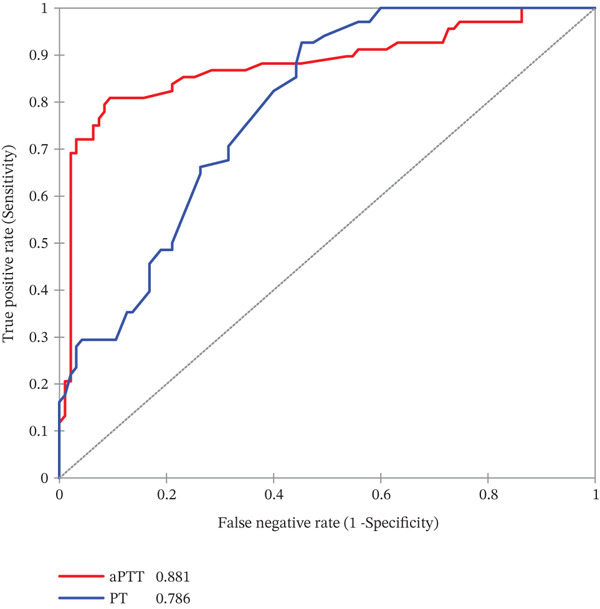
(a)

**Figure:**
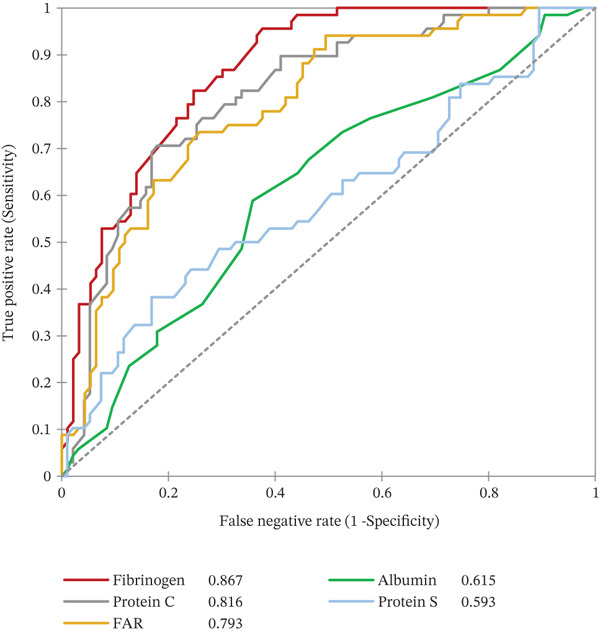
(b)

### 3.7. Diagnostic Performance of aPTT, PT, Fibrinogen, Protein C, FAR, Albumin, and Protein S as Potential Indicator of Chronic Viral Hepatitis B Infection

At a cutoff of ≥ 35.7 s, aPTT was the best measure for indicating chronic HBV infection with a sensitivity of 79.4% and a specificity of 91.6%, whereas PT and fibrinogen were both highly sensitive (92.6% and 95%) but less specific (54.7% and 62.4%), respectively. However, albumin and protein S were less sensitive in indicating chronic viral hepatitis B infection (Table 3).

**Table 3:** Diagnostic performance of aPTT, PT, fibrinogen, protein C, FAR, albumin, and protein S as potential indicators of chronic viral hepatitis B infection.

Parameters	Cutoffs	Sensitivity (%)	Specificity (%)	PPV (%)	NPV (%)	LR+	Accuracy
aPTT (secs)	≥ 35.7	79.4	91.6	87.1	86.1	9.4	86.5
PT (secs)	≥ 14.5	92.6	54.7	59.4	91.2	2	70.6
Fibrinogen (g/dL)	≤ 6.17	95.6	62.4	65	95.1	2.5	76.4
Protein C (*μ*g/mL)	≤ 2.55	70.6	82.1	73.8	79.6	3.9	77.3
FAR	≤ 0.16	73.5	74.2	67.6	79.3	2.8	73.9
Albumin (g/dL)	≤ 33.0	58.8	64.2	54.1	68.5	1.6	62.0
Protein S (*μ*g/mL)	≤ 2.31	50.1	89.4	61.9	65.3	2.3	64.4

Abbreviations: aPTT, activated partial thromboplastin time; FAR, fibrinogen/albumin ratio; LR+, positive likelihood ratio; NPV, negative predictive value; PPV, positive predictive value; PT, prothrombin time.

## 4. Discussion

Viral hepatitis infection is a major risk factor for adverse fetal‐maternal complications during pregnancy. Both the acute and chronic forms of viral hepatitis have been associated with impaired liver function thereby altering the hemostatic balance. Pregnancy also presents with physiological changes in the hemostatic balance resulting in a shift towards a state of increased blood clotting tendency. However, studies on coagulation disturbances in pregnant women with concurrent viral hepatitis B infection are lacking. Hence, this study assessed the coagulation profile among hepatitis B‐infected pregnant women in the Ghanaian population.

As far as sociodemographic characteristics are concern; age, gender, marital status, and employment status were similar among cases and controls, which agrees with a study conducted by Anaedobe et al. [ 19 ]. However, alcohol consumption and contraceptive use were statistically different in the hepatitis B patients and controls. In addition to this revelation, the number of individuals who use contraceptives were high in the HBV group as compared to the controls, a similar trend was seen in a study done by Anaedobe et al. [19]. Bayo et al. and Onwuiri et al. reported that behavioral factors including risky sexual acts may be more prevalent among contraceptive users, which indirectly increases the risk of transmission [20, 21]. Alcohol consumption has been shown to impair the function of the immune system, and this may increase the susceptibility of consumers to infection [20, 22].

In this present study, the increased levels of AST, ALP, GGT, bilirubin (total, direct, and indirect), and decreased levels of albumin were observed in the HBV‐infected pregnant women compared with controls. These findings are consistent with previous studies conducted by Onwuiri et al. and Abulude et al. who showed that HBsAg seropositivity was associated with high prevalence of abnormal ALP, AST, total, and direct bilirubin [11, 21]. The AST levels was significantly different in the various groups especially in the chronic HBV group and the controls. The persistence of elevated AST levels in chronic infection may suggest continuous, low‐grade liver inflammation. In contrast, ALT levels did not differ significantly among the study groups. Although ALT is generally considered a more specific indicator of hepatocellular injury, its levels can fluctuate according to the phase of HBV infection and the host immune response [23, 24].

In this study, HBV‐infected pregnant women presented with prolonged PT, aPTT, and INR compared with controls. This finding is consistent with previous studies by Olley et al. and Xu et al., which found increased values of these tests in HBV‐infected patients compared with controls [25, 26]. Studies have reported that viral liver infection results in the production of tumor necrosis factor (TNF), which contributes to liver pathology and which can lead to impaired liver synthetic function [27–29]. This leads to a decrease in the procoagulant factors of both the intrinsic and extrinsic pathways, which disrupts the coagulation cascade and prolongs the test times [30, 31].

The levels of fibrinogen were decreased in both chronic and acute viral infections compared with controls. This finding is similar to reports by Xu et al. [26] who found decreased levels of fibrinogen in HBV positive patients compared with controls. Fibrinogen is primarily synthesized in the liver, and as a positive phase reactant protein, its levels rise during inflammation [32]. Physiological changes in pregnancy also lead to an increase in the levels of fibrinogen levels [33, 34]. Nonetheless, hepatitis B infection results in an impaired synthetic function of the liver, leading to decrease fibrinogen levels [35]. Moreover, the level of FAR was decreased in both the hepatitis groups than controls. Albumin‐likewise fibrinogen is mainly synthesized in the liver; hence, its dysfunction decreases the levels of these markers. This is inconsistent with previous studies that showed that elevated levels of FAR can serve as a valuable predictor of severity and likelihood of death of various clinical conditions, including cancer, cardiovascular diseases, and cerebrovascular disorders [36–38].

Both protein C and protein S serve as natural anticoagulants and, like the above, are primarily synthesized in the liver; hence, a dysfunctional liver impairs it synthesis. In our study, protein C levels were decreased in pregnant women with chronic and acute infection compared with controls, whereas protein S was only decreased in women with chronic viral hepatitis. Previous studies have reported decreased protein C levels in hepatitis‐positive patients compared with controls, which is consistent to our study [39, 40]. In contrast, Güven et al. [41] observed comparable protein C and protein S levels in patients with chronic hepatitis compared with controls. These inconsistencies may be explained by the differences in sample size and study population.

In the regression analysis, fibrinogen levels did not show any significant association with both PT and aPTT. In contrast, a previous study by Leticia et al. [7] showed a statistically significant positive relationship between serum fibrinogen levels and the coagulation test times, PT and aPTT. However, this study was conducted among hepatitis‐infected blood donors while our study population employed pregnant women and this may account for the differences.

Finally, the ROC analysis suggests that aPTT, fibrinogen, and protein C could help identify chronic HBsAg‐positive pregnant women at higher risk of hemostatic abnormalities, providing a potential means of monitoring disease severity. Interestingly, aPTT, fibrinogen, and protein C had excellent diagnostic accuracy, and thus may be valuable indicators for mitigating chronic hepatitis‐related complications including cirrhosis, hepatocellular carcinoma, and liver failure. PT and FAR also showed better diagnostic accuracy but were inferior to the above indices, whereas protein S and albumin showed the poorest diagnostic accuracy. Peng et al. [42] also observed that aPTT, PT, and fibrinogen had high AUCs and were excellent indicators for early diagnosis of cirrhosis. Although HBsAg testing remains the primary diagnostic tool for HBV infection, aPTT, fibrinogen, and protein C may serve as useful indicators of chronic HBV‐related liver dysfunction or coagulopathy.

The strength of this study lies in being the first case–control investigation to simultaneously assess the hematobiochemical and coagulation profiles among HBV‐infected pregnant women. Notwithstanding these novel findings, several limitations should be considered for future research. First, the study did not include trimester‐specific or gestational‐age–based analyses of laboratory parameters; consequently, potential physiological variations across different stages of pregnancy could not be assessed. Future studies should incorporate gestational‐age–specific evaluations to provide a more comprehensive understanding of laboratory changes during pregnancy. Furthermore, the possible use of conventional or nonconventional treatments (e.g., herbal remedies) or occupational exposure to toxins prior to the study could have affected liver enzyme levels, but these factors were not assessed.

Other coagulation markers, such as prothrombin fragment F1 and thrombin–antithrombin complexes, were not measured; their inclusion could provide deeper insights into coagulation abnormalities in HBV‐infected pregnancies. The study was also conducted at a single center, which may limit the generalizability of the findings to the broader Ghanaian population. Finally, the small sample size of participants with acute HBV infection (*n* = 14) limits the statistical power and reliability of subgroup comparisons, and these findings should therefore be interpreted with caution. Larger, multicenter studies are needed to validate these observations.

## 5. Conclusion

Pregnant women with HBV infection present with significant increases in AST, ALP, GGT, and bilirubin levels. Also, HBV causes significant changes in coagulation parameters and natural anticoagulants; however, these alterations are generally comparable in acute and chronic hepatitis B infection. aPTT, fibrinogen, and protein C showed excellent diagnostic accuracy as indicators of chronic HBV‐related infection, and thus may be valuable supportive indicators in managing chronic hepatitis‐related complications. The study, therefore, concludes that pregnant women with HBV infection have higher levels of AST, GGT, ALP, prolonged PT, and aPTT with reduced levels of protein C, protein S, and fibrinogen. Implementing therapeutic strategies focused on preserving the functionality of the coagulation system can significantly improve supportive care for pregnant women with hepatitis B virus.

**Nomenclature**
aPTT: activated partial thromboplastin time
ALT: alanine aminotransferase
ALP: alkaline phosphatase
AST: aspartate aminotransferase
AT: antithrombin
AUC: area under the curve
CTLs: cytotoxic T lymphocytes
DVT: deep vein thrombosis
DNA: deoxyribonucleic acid
EDTA: ethylenediaminetetraacetic acid
ELISA: enzyme‐linked immunosorbent assay
FAR: fibrinogen to albumin ratio
GGT: gamma glutamyl transferase
G6PD: glucose 6‐phosphate deficiency dehydrogenase
HBV: hepatitis B virus
HCV: hepatitis C virus
HBcAb: hepatitis B core antibody
HBeAg: hepatitis B e‐antigen
HBIG: hepatitis B immunoglobulin
HBsAb: hepatitis B surface antibody
HBsAg: hepatitis B surface antigen
HRP: horseradish peroxidase
INR: international normalized ratio
LFT: liver function test
LR+: positive likelihood ratio
MTCT: mother‐to‐child‐transmission
NPV: negative predictive value
PPP: platelet‐poor plasma
PPV: positive predictive value
PT: prothrombin time
ROC: receiver operating characteristics
RBC: red blood cell
RDW: red blood cell distribution width
RNA: ribonucleic acid
t‐PA: tissue plasminogen activator
VTE: venous thromboembolism
WBC: white blood cell count
WHO: World Health Organization

## Author Contributions

Conceptualization: A.A.K., W.I.O.B., E.O.A. Data curation: A.A.K., W.I.O.B., A.A.A., S.T., D.A.A. Formal analysis: J.F., S.T., A.A.A. Investigation: A.A.K., W.I.O.B., F.A.A, A.A.A., S.T., D.A.A. Methodology: A.A.K., W.I.O.B., E.O.A., S.T., A.A.A. Project administration: A.A.K., W.I.O.B., E.O.A. Validation: A.A.K., W.I.O.B., E.O.A. Visualization: A.A.K., W.I.O.B., F.A.A., A.A.A., S.T., D.A.A. Writing – original draft: A.A.K., W.I.O.B., F.A.A., A.A.A., S.T., D.A.A., E.A., A.N.B., D.N.M.A., E.O.A. Writing – review, editing, and approving: All authors.

## Funding

No funding was received for this manuscript.

## Disclosure

A preprint has previously been published [18].

## Ethics Statement

Ethical approval was obtained from the Committee on Human Research, Publication and Ethics, School of Medical Sciences, Kwame Nkrumah University of Science and Technology (CHRPE/AP/834/22), as well as the management of Afrancho Polyclinic. The ethical approval was fixed for 1 year from 19th December, 2022, to 18th December, 2023.

## Consent

Both oral and written consent from all participants was obtained before their recruitment. All participants had the age to consent.

## Conflicts of Interest

The authors declare no conflicts of interest.

## Data Availability

Data and materials for study are available upon request from the corresponding authors.

## References

[1] World Health Organization , Global Progress Report on HIV, Viral Hepatitis and Sexually Transmitted Infections, 2021, 2021, Accountability for the Global Health Sector Strategies 2016–2021: Actions for Impact: Web Annex 1: Key Data at a Glance. Global Progress Report on HIV, Viral Hepatitis and Sexually Transmitted Infections, 2021: Accountability for the Global Health Sector Strategies 2016–2021: Actions for Impact: Web Annex 1: Key Data at a Glance 2021.

[2] Pan X. , Chen J. , Zhou L. , Ou X. , He F. , Liu Y. , Zheng S. , Wang H. , Cao B. , Wang Z. , Liu H. , Liu G. , Huang Z. , Shen G. , Liu S. , and Chen D. , Efficacy and Safety of Continuous Antiviral Therapy From Preconception to Prevent Perinatal Transmission of Hepatitis B Virus, Scientific Reports. (2020) 10, no. 1, 13631, 10.1038/s41598-020-70644-4, 32788743. 32788743 PMC7423885

[3] Alemu A. A. , Zeleke L. B. , Aynalem B. Y. , and Kassa G. M. , Hepatitis B Virus Infection and Its Determinants Among Pregnant Women in Ethiopia: A Systematic Review and Meta-Analysis, Infectious Diseases in Obstetrics and Gynecology. (2020) 2020, 9418475, 10.1155/2020/9418475, 32577077. 32577077 PMC7305536

[4] Ukagebu C. J. , Alao J. O. , Bamigboye F. O. , Ukaegbu J. C. , and Oladipo E. K. , Evaluating Hepatitis B Screening During Pregnancy: A Study on Diagnostic Accuracy and Infection Control in Nigeria, Journal of Viral Hepatitis. (2025) 32, no. 2, e70002, 10.1111/jvh.70002, 39831579. 39831579 PMC11744738

[5] Sinha S. and Kumar M. , Pregnancy and Chronic Hepatitis B Virus Infection, Hepatology Research. (2010) 40, no. 1, 31–48, 10.1111/j.1872-034X.2009.00597.x, 2-s2.0-77954361430, 20156298. 20156298

[6] Majerus P. W. and Tollefsen D. M. , Brunton L. , Blood Coagulation and Anticoagulant, Thrombolytic, and Antiplatelet Drugs, Goodman and Gilman′s the Pharmacological Basis of Therapeutics, 2006, 11th edition, McGraw-Hill, 1467–1488.

[7] Leticia O. I. , Andrew A. , Ifeanyi O. E. , Ifeoma U. E. , and Ugochukwu A. , The Effect of Viral Hepatitis on APTT, PT, TT, Fibrinogen and Platelet Among Blood Donors at FMC, Umuahia, IOSR Journal of Dental and Medical Sciences. (2014) 13, 57–63.

[8] Pant A. , Kopec A. K. , and Luyendyk J. P. , Role of the Blood Coagulation Cascade in Hepatic fibrosis, Physiology. (2018) 315, no. 2, G171–G176, 10.1152/ajpgi.00402.2017, 2-s2.0-85051671550. PMC6139645 29723040

[9] Hyers T. M. , Agnelli G. , Hull R. D. , Morris T. A. , Samama M. , Tapson V. , and Weg J. G. , Antithrombotic Therapy for Venous Thromboembolic Disease, Chest. (2001) 119, no. 1, 176S–193S, 10.1378/chest.119.1_suppl.176S, 2-s2.0-0035125568. 11157648

[10] Ryan J. M. and Heneghan M. A. , Pregnancy and the Liver, Clinical Liver Disease. (2014) 4, no. 3, 51–54, 10.1002/cld.361, 2-s2.0-84908672862.

[11] Abulude O. A. , Ahmed I. , and Sadisu F. U. , Assessment of Hepatitis B Viral Infection as a Predictor of Hepatic Enzymes and Compounds Alteration Among Antenatal Patients, Medical Science. (2017) 5, no. 4, 10.3390/medsci5040024. PMC5753653 29099040

[12] Sirilert S. and Tongsong T. , Hepatitis B Virus Infection in Pregnancy: Immunological Response, Natural Course and Pregnancy Outcomes, Journal of Clinical Medicine. (2021) 10, no. 13, 10.3390/jcm10132926. PMC8267880 34210105

[13] King J. and Westbrook R. , Pregnancy and Liver Disease, Evidence-Based Gastroenterology and Hepatology 4e, 2019, Wiley, 408–424.

[14] Lampertico P. , Agarwal K. , Berg T. , Buti M. , Janssen H. L. , Papatheodoridis G. , Zoulim F. , and Tacke F. , EASL 2017 Clinical Practice Guidelines on the Management of Hepatitis B Virus Infection, Journal of Hepatology. (2017) 67, no. 2, 370–398, 10.1016/j.jhep.2017.03.021, 2-s2.0-85017533115. 28427875

[15] Adjei C. A. , Atibila F. , Apiribu F. , Ahordzor F. , Attafuah P. A. , Ansah-Nyarko M. , Asamoah R. , and Menkah W. , Hepatitis B Infection Among Parturient Women in Peri-Urban Ghana, American Journal of Tropical Medicine and Hygiene. (2018) 99, no. 6, 1469–1474, 10.4269/ajtmh.17-0752, 2-s2.0-85058194198, 30298807. 30298807 PMC6283520

[16] Gish R. G. , Basit S. A. , Ryan J. , Dawood A. , and Protzer U. , Hepatitis B Core Antibody: Role in Clinical Practice in 2020, Current Hepatology Reports. (2020) 19, no. 3, 254–265, 10.1007/s11901-020-00522-0.

[17] Kim W. R. , Berg T. , Asselah T. , Flisiak R. , Fung S. , Gordon S. C. , Janssen H. L. , Lampertico P. , Lau D. , Bornstein J. D. , Schall R. E. , Dinh P. , Yee L. J. , Martins E. B. , Lim S. G. , Loomba R. , Petersen J. , Buti M. , and Marcellin P. , Evaluation of APRI and FIB-4 Scoring Systems for Non-Invasive Assessment of Hepatic Fibrosis in Chronic Hepatitis B Patients, Journal of Hepatology. (2016) 64, no. 4, 773–780, 10.1016/j.jhep.2015.11.012, 2-s2.0-84957066037, 26626497. 26626497

[18] Khalifah A. A. , Boadu W. I. , Amponsah F. A. , Frimpong J. , Ayirebi A. A. , Twumasi S. , Afrifah D. A. , Appiagyei E. , Boadu A. N. , Antonio D. N. , and Anto E. O. , The effect of Hepatitis B Viral Infection on Hemostatic Profile and Liver Markers in Ghanaian Pregnant Women, 2025, 10.21203/rs.3.rs-7849679/v1.

[19] Anaedobe C. G. , Fowotade A. , Omoruyi C. E. , and Bakare R. A. , Prevalence, Sociodemographic Features and Risk Factors of Hepatitis B Virus Infection Among Pregnant Women in Southwestern Nigeria, Pan African Medical Journal. (2015) 20, 10.11604/pamj.2015.20.406.6206, 2-s2.0-84940183678. PMC4524914 26301010

[20] Bayo P. , Ochola E. , Oleo C. , and Mwaka A. D. , High Prevalence of Hepatitis B Virus Infection Among Pregnant Women Attending Antenatal Care: A Cross-Sectional Study in Two Hospitals in Northern Uganda, BMJ Open. (2014) 4, no. 11, e005889, 10.1136/bmjopen-2014-005889, 2-s2.0-84911998115, 25387757. PMC4244481 25387757

[21] Onwuiri F. , Ndako J. A. , and Onwuliri E. , Prevalence of Hepatitis B Virus (HBV) and Hepatitis C Virus (HCV) and Their Effects on Serum Albumin and Liver Aminotransferases in Pregnant Women in Jos, Virology: Research and Reviews. (2017) 1, no. 2, 10.15761/VRR.1000108.

[22] Saleh D. , Prevalence and Risk Factors of HBV Infection Among Pregnant Women in Urban and Rural Egyptian Communities, Journal of Liver. (2015) 4, no. 3.

[23] Kumar M. , Satapathy S. , Monga R. , Das K. , Hissar S. , Pande C. , Sharma B. C. , and Sarin S. K. , A Randomized Controlled Trial of Lamivudine to Treat Acute Hepatitis B, Hepatology. (2007) 45, no. 1, 97–101, 17187417, 10.1002/hep.21486, 2-s2.0-33846447769. 17187417

[24] Terrault N. A. , Lok A. S. , McMahon B. J. , Chang K. M. , Hwang J. P. , Jonas M. M. , Brown RS Jr , Bzowej N. H. , and Wong J. B. , Update on Prevention, Diagnosis, and Treatment of Chronic Hepatitis B: AASLD 2018 Hepatitis B Guidance, Hepatology. (2018) 67, no. 4, 1560–1599, 10.1002/hep.29800, 2-s2.0-85044252801, 29405329. 29405329 PMC5975958

[25] Olley M. , Zekeri S. , Erhamwonyi A. K. , Okwu M. U. , Osayomwanbo A. D. , Clement O. O. , and Ekundayo I. O. , Coagulation Parameters Among Individuals With Hepatitis B Infections in Okada, Edo State, Nigeria, Journal of Advances in Medical and Pharmaceutical Sciences. (2023) 25, no. 1, 44–49, 10.9734/jamps/2023/v25i1597.

[26] Xu H.-Q. , Wang C.-G. , Zhou Q. , and Gao Y.-H. , Effects of Alcohol Consumption on Viral Hepatitis B and C, World Journal of Clinical Cases. (2021) 9, no. 33, 10052–10063, 10.12998/wjcc.v9.i33.10052, 34904075. 34904075 PMC8638036

[27] Herbein G. and O′brien W. A. , Tumor Necrosis Factor (TNF)–*α* and TNF Receptors in Viral Pathogenesis (44487), Proceedings of the Society for Experimental Biology and Medicine: Minireviews. (2000) 223, no. 3, 241–257, 10.1177/153537020022300305. 10719836

[28] Orange J. S. , Salazar-Mather T. P. , Opal S. M. , and Biron C. A. , Mechanisms for Virus-Induced Liver Disease: Tumor Necrosis Factor-Mediated Pathology Independent of Natural Killer and T Cells During Murine Cytomegalovirus Infection, Journal of Virology. (1997) 71, no. 12, 9248–9258, 9371583. 9371583 10.1128/jvi.71.12.9248-9258.1997 PMC230227

[29] Tiegs G. and Horst A. K. , TNF in the Liver: Targeting a Central Player in Inflammation, Seminars in Immunopathology. (2022) 44, no. 4, 445–459, 10.1007/s00281-022-00910-2. 35122118 PMC9256556

[30] Hoffman M. , Coagulation in Liver Disease, Seminars in Thrombosis and Hemostasis. (2015) 41, no. 5, 447–454, 10.1055/s-0035-1550435, 2-s2.0-84937815333. 26049068

[31] Kenny L. , Baker P. N. , and Cunningham F. G. , Platelets, Coagulation, and the Liver, Chesley′s Hypertensive Disorders in Pregnancy, 2009, Academic Press, 335–351, 10.1016/B978-0-12-374213-1.00018-5, 2-s2.0-84882467878.

[32] Ghanim B. , Hoda M. , Klikovits T. , Winter M. , Alimohammadi A. , Grusch M. , Dome B. , Arns M. , Schenk P. , Jakopovic M. , Samarzija M. , Brcic L. , Filipits M. , Laszlo V. , Klepetko W. , Berger W. , and Hegedus B. , Circulating Fibrinogen Is a Prognostic and Predictive Biomarker in Malignant Pleural Mesothelioma, British Journal of Cancer. (2014) 110, no. 4, 984–990, 10.1038/bjc.2013.815, 2-s2.0-84894307206, 24434429. 24434429 PMC3929892

[33] Brenner B. , Haemostatic Changes in Pregnancy, Thrombosis Research. (2004) 114, no. 5-6, 409–414, 10.1016/j.thromres.2004.08.004, 2-s2.0-7044249206, 15507271. 15507271

[34] Deitcher S. R. and Gardner J. F. , Physiologic Changes in Coagulation and Fibrinolysis During Normal Pregnancy, Clinics in Liver Disease. (1999) 3, no. 1, 83–96, 10.1016/S1089-3261(05)70055-6, 2-s2.0-85013272353.

[35] Papadopoulos V. , Filippou D. , Manolis E. , and Mimidis K. , Haemostasis Impairment in Patients With Obstructive Jaundice, Journal of Gastrointestinal and Liver Diseases. (2007) 16, no. 2, 177–186, 17592568. 17592568

[36] Çetin M. , Erdoğan T. , Kırış T. , Özer S. , Yılmaz A. , Durak H. , Aykan A. Ç. , and Şatıroğlu Ö. , Predictive Value of Fibrinogen-to-Albumin Ratio in Acute Coronary Syndrome, Herz. (2020) 45, no. Supplement 1, 145–151, 10.1007/s00059-019-4840-5, 2-s2.0-85070210694, 31388710. 31388710

[37] Liu G. , Fan C.-M. , Guo H. , Fan W.-N. , Li M.-L. , and Cui G.-X. , Fibrinogen-to-Albumin Ratio Predicts Long-Term Outcomes for Patients With ST-Elevation Myocardial Infarction and Multivessel Disease: A Prospective Observational Cohort Study, Experimental and Therapeutic Medicine. (2021) 21, no. 5, 10.3892/etm.2021.9896, 33767762. PMC7976379 33767762

[38] Sun F. , Tan Y. A. , Gao Q. F. , Li S. Q. , Zhang J. , Chen Q. G. , Jiang Y. H. , Zhang L. , Ying H. Q. , and Wang X. Z. , Circulating Fibrinogen to Pre-Albumin Ratio Is a Promising Biomarker for Diagnosis of Colorectal Cancer, Journal of Clinical Laboratory Analysis. (2019) 33, no. 1, e22635, 10.1002/jcla.22635, 2-s2.0-85060211628. 30047185 PMC6430345

[39] Saja M. F. , Abdo A. A. , Sanai F. M. , Shaikh S. A. , and Gader A. G. M. A. , The Coagulopathy of Liver Disease: Does Vitamin K Help?, Blood Coagulation & Fibrinolysis. (2013) 24, no. 1, 10–17, 10.1097/MBC.0b013e32835975ed, 2-s2.0-84871800702, 23080365. 23080365

[40] Saray A. , Mesihovic R. , Vanis N. , Gornjakovic S. , and Prohic D. , Clinical Significance of Haemostatic Tests in Chronic Liver Disease, Medical Archives. (2012) 66, no. 4, 231–235, 22919876, 10.5455/medarh.2012.66.231-235, 2-s2.0-84867058683. 22919876

[41] Gürsoy Ş. , Başkol M. , Torun E. , Yurci A. , Soyuer I. , Eser B. , Güven K. , Özbakir Ö. , and Yücesoy M. , Importance of Anticoagulant Proteins in Chronic Liver Diseases, Turkish Journal of Gastroenterology. (2005) 16, no. 3, 129–133, 16245221. 16245221

[42] Peng J. , He G. , Chen H. , and Kuang X. , Study on Correlation Between Coagulation Indexes and Disease Progression in Patients With Cirrhosis, American Journal of Translational Research. (2021) 13, no. 5, 4614–4623, 34150041. 34150041 PMC8205686

